# The Poplar-Poplar Rust Interaction: Insights from Genomics and Transcriptomics

**DOI:** 10.4061/2011/716041

**Published:** 2011-10-26

**Authors:** Stéphane Hacquard, Benjamin Petre, Pascal Frey, Arnaud Hecker, Nicolas Rouhier, Sébastien Duplessis

**Affiliations:** Institut National de la Recherche Agronomique (INRA), Nancy Université, Unité Mixte de Recherche 1136, “Interactions Arbres/Micro-organismes,” Centre INRA de Nancy, 54280 Champenoux, France

## Abstract

Poplars are extensively cultivated worldwide, and their susceptibility to the leaf rust fungus *Melampsora larici-populina* leads to considerable damages in plantations. Despite a good knowledge of the poplar rust life cycle, and particularly the epidemics on poplar, the perennial status of the plant host and the obligate biotrophic lifestyle of the rust fungus are bottlenecks for molecular investigations. Following the completion of both *M. larici-populina* and *Populus trichocarpa* genome sequences, gene families involved in poplar resistance or in rust fungus virulence were investigated, allowing the identification of key genetic determinants likely controlling the outcome of the interaction. Specific expansions of resistance and defense-related genes in poplar indicate probable innovations in perennial species in relation with host-pathogen interactions. The genome of *M. Larici-populina* contains a strikingly high number of genes encoding small secreted proteins (SSPs) representing hundreds of candidate effectors. Transcriptome analyses of interacting partners in compatible and incompatible interactions revealed conserved set of genes involved in poplar defense reactions as well as timely regulated expression of SSP transcripts during host tissues colonisation. Ongoing functional studies of selected candidate effectors will be achieved mainly on the basis of recombinant protein purification and subsequent characterisation.

## 1. The *Populus/Melampsora* Interaction

Poplars are fast growing trees naturally present in riparian forests of the northern hemisphere [1]. Hybrid poplars are extensively cultivated worldwide for wood production and have recently received growing attention for bioenergy research programs [2]. During the last decades, breeders have generated a collection of hybrid poplars with complete resistances to *M. larici-populina*. Nevertheless, culture practices in monoclonal plantations enhanced the rapid breakdown of selected poplar resistances [1]. Nowadays, almost all poplar cultivars are susceptible to the rust fungus, and dramatic damages are observed in plantations [1]. Thus, *M. larici-populina* represents the major threat of poplar in plantations, and it is crucial to identify key determinants controlling the outcome of the poplar-poplar rust interaction in order to define new strategies to contain the disease. In addition, the study of this pathosystem should also provide new insights into the molecular mechanisms associated with fungal biotrophy and host resistance in perennial plants.

**Figure 1:**
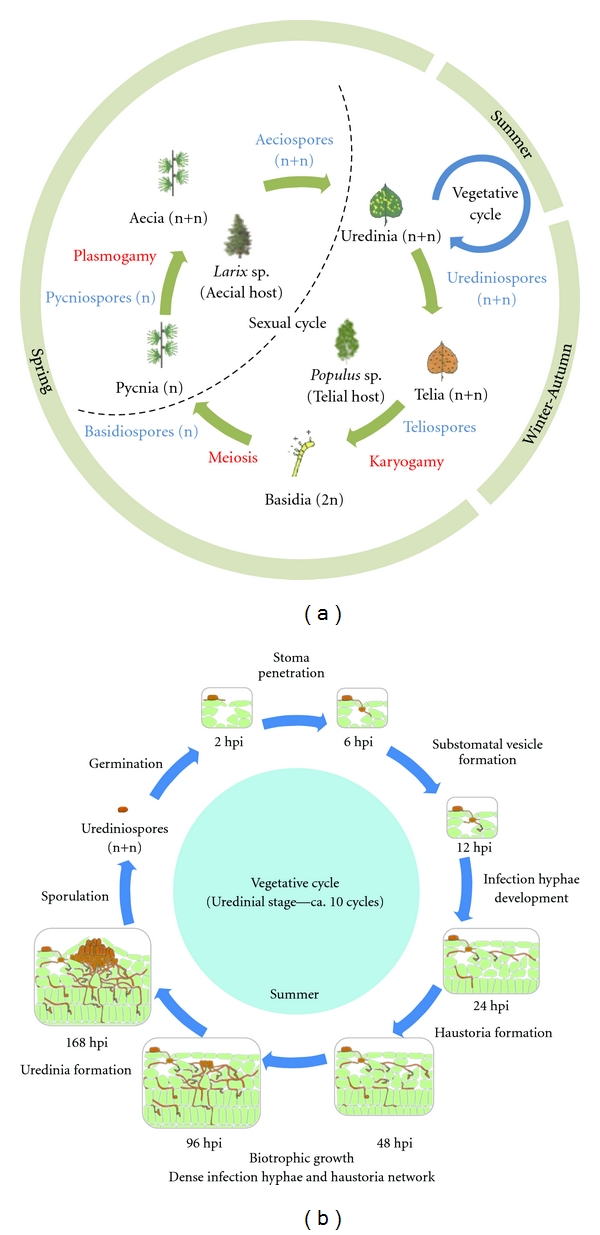
Life cycle of *Melampsora larici-populina*. (a) Biological macrocyclic heteroecious cycle of *M. larici-populina*. (b) Vegetative cycle occurring on poplar leaves and used as a model for molecular investigations of the poplar-poplar rust interaction.

*M. larici-populina* is a basidiomycete biotroph pathogen belonging to the Pucciniales order (Pucciniomycotina, Pucciniomycetes, Pucciniales, Melampsoraceae). This leaf rust fungus has a complex heteroecious macrocyclic lifestyle; that is, the biological cycle is completed on two different hosts and implies five different spore forms (detailed in Figure 1(a)). In early spring, overwintered diploid teliospores (2n) that have undergone karyogamy and meiosis in ground decaying poplar leaves (telial host) produce haploid basidiospores (n). After dissemination by the wind, these spores achieve a single infection on larch (aecial host) needles leading to the production of pycniospores (n). Fusion of opposite mating types generates aecia and dikaryotic aeciospores (n+n, sexual phase). These wind-borne spores then infect poplar leaves and differentiate another sporulation structure called the uredinium, which corresponds to an orange pustule formed on the abaxial epidermis of mature leaves, the typical symptom of the disease on poplar trees. Large amounts of urediniospores (n+n, asexual phase) are released from uredinia and dispersed over very large distances [3, 4]. Several vegetative infection cycles can be completed on poplar leaves during spring and summer. In autumn, black telia pustules containing teliospores (n+n) are produced in senescent poplar leaves.

Since damages observed in poplar plantations occur during the asexual development of the rust fungus (detailed in Figure 1(b)), this specific phase has received attention from several research groups focusing on different aspects of the poplar-poplar rust interaction (for review, see [1, 5]). The major developmental transitions of the fungus (i.e., differentiated infection cell types) have been described by microscopy during colonisation of poplar leaves in controlled experimental conditions [6–9]. First, urediniospores (n+n) germinate on the abaxial surface of poplar leaves and produce germ tubes that penetrate through stomata within the first 6 hours postinoculation (hpi). After 12 hpi, substomatal vesicles are formed in the spongy mesophyll, from which infection hyphae extend into the mesophyll and differentiate the first haustorial structures as soon as 17 hpi [6]. In the case of a compatible interaction, biotrophic growth goes on and the fungal biomass strongly increases between 48 and 96 hpi (i.e., >30-fold) [7, 10], forming a dense network of infection hyphae and haustoria in the mesophyll nearby primary infection sites [7]. Around seven days after inoculation (168 hpi), fungal pressure generates a breach in the abaxial epidermis and leads to the formation of uredinia releasing newly formed urediniospores at the surface of the leaves [7, 9]. In the case of an incompatible interaction, fungal growth is arrested early during the colonisation process, concomitant with strong plant defense reactions [6, 7, 10]. Cytological observations revealed a highly localized hypersensitive response (HR), with collapsed infected plant cells and accumulation of monolignols around infection sites after 48 hpi in the incompatible interaction [6, 7]. At later time points in the compatible interaction, anthocyanidins, lignin, pectin, and hydrogen peroxide accumulate around infection sites and likely participate in late defense responses and partial resistance in poplar [5, 8, 11]. 

In spite of the efforts initiated to describe this pathosystem, the obligate biotrophic status of *Melampsora* spp., the lack of efficient systems for genetic transformation of hybrid poplars susceptible to the rust fungus, and the long generation time of poplar all together represent a serious bottleneck for molecular investigations. Fortunately, recent advances in tree and fungal genomics have helped in defining new strategies to facilitate the study of this tree-rust fungus model pathosystem. Indeed, the genome sequence of the black cottonwood *Populus trichocarpa* “Nisqually-1” was the first tree genome sequenced by the Joint Genome Institute (JGI, Department of Energy, USA) [12]. As part of a community-sequencing project aiming to decipher lifestyles of poplar microbiome, the genome of *M. larici-populina* (strain 98AG31) has been sequenced by the JGI along with those of symbiotic fungi interacting with poplar roots [13, 14]. The genome sequence of the poplar rust fungus has been recently released [15]. 

The availability of both the host and the parasite genome sequences offers unparalleled opportunities to study gene families involved in plant defense and pathogen virulence within an integrated pathosystem [5, 16, 17]. Comparative genomic studies with other plants and biotrophic fungi or oomycetes also help to decipher the evolutionary trends underlying plant-pathogen interactions in perennial plant species [18]. The access to these reference genomes is also a great opportunity to perform transcriptome analyses through the use of whole-genome custom exon oligoarrays or high-throughput sequencing technologies (RNA-Seq).

## 2. Learning from the Genomes of Poplar and *M. larici-populina*

With the complete genome sequences of *P. trichocarpa* and *M. larici-populina*, performing *in silico *gene family analyses is a critical step to decipher expression, evolution, and biological functions of genes and proteins participating or regulating a wide variety of mechanisms related to plant immunity or fungal pathogenesis. 

Genome-wide analyses of poplar gene families previously reported to be related to pathogen response in plants have been summarized in Table 1, taking as a basis a previous report by Yang and collaborators in 2009 [16]. Globally, researches were mostly devoted to the study of functions such as secondary metabolism associated with plant cell wall and wood formation, hormone biosynthesis, transcription factors, signalling pathways, and redox homeostasis (Table 1). Considering the flow of data available, here we essentially focus on analyses performed from the standpoint of the poplar-poplar rust pathosystem. Kohler and collaborators [19] reported in 2008 the genome-wide analysis of poplar genes coding for nucleotide-binding leucine-rich repeat (NB-LRR) proteins, representing a large class of plant resistance genes (*R* genes) responsible for pathogen effector recognition and leading to complete resistance through effector-triggered immunity in many pathosystems [20, 21]. Approximately 400 *NB-LRR* genes were identified in the genome of *P. trichocarpa*, which is twice larger than *NB-LRR* genes reported in *Arabidopsis thaliana* (402 versus 178, resp.). The presence of about 500 *NB-LRR* genes in the rice genome does not support a specific increase of this gene family in perennial species [5]; however, the content in *NB-LRR *gene classes (TIR-NB-LRR and non-TIR-NB-LRR) differs between monocot and dicot genomes [19]. Interestingly, many of the poplar *NB-LRR* genes (more than 70 according to [22]) are gathered into a supercluster localized on the chromosome 19, in the neighbourhood of many transposable elements (retrotransposons). Since retroelements are known to impact gene family increase and diversification, it is tempting to hypothesize that this supercluster is likely a nursery for new poplar *R* genes [23]. Recently, the fine mapping of two rust resistance loci associated with complete resistance and partial resistance to *M. larici-populina *was achieved on the peritelomeric end of the chromosome 19 in the *NB-LRR *genes supercluster, which strongly suggests that rust-related *R *genes belong to the NB-LRR class [22]. In addition to the coiled-coil (CC) and toll-interleukine receptor (TIR) NB-LRR classes, a third class of *NB-LRR* genes containing a BED finger domain (called BED-NB-LRR, henceforth called BNL) has also been reported in the poplar genome [19, 24]. The discovery of the BED domain was first published in 2000 and was termed BED finger, after two *Drosophila* proteins named BEAF and DREF containing this domain [25]. The BED motif corresponds to a ubiquitous zinc-finger DNA-binding domain, raising the possible involvement of such BNL proteins in interaction with DNA and eventually regulation of transcription, although no evidence for such mechanism has been provided yet [24]. Upon recognition of a pathogen, plant R proteins trigger defence response reactions, marked by the strong induction of pathogenesis-related proteins (PR proteins) at the transcript and protein levels [26]. Interestingly, in addition to the large number of *R* genes, the poplar genome also contains an expanded gene family encoding thaumatin-like proteins (TLPs), corresponding to the PR-5 proteins. This gene family illustrates an overrepresentation of defense-related genes in poplar compared with annual species with 42 genes identified in *P. trichocarpa* versus 22 in the *A. thaliana* genome sequence [12, 27]. A study conducted on a total of 600 TLPs retrieved from 100 species in international databases revealed a cluster of phylogenetically related TLPs, enriched in poplar and tree sequences, that might represent a specific innovation in perennial species. Beyond the dramatic expansion of these defense-related genes in the poplar genome, diversification and subsequent sub- or neo-functionalisation likely occurred in poplar TLPs as exemplified by diversifying selection observed in this cluster [27, 28]. So far, other PR gene families have not been explored at the genome-wide scale in poplar, and it would be interesting to determine whether other expansions of specific gene family related to plant immunity occurred in trees, representing possible innovations in these long-living species.

**Figure 2:**
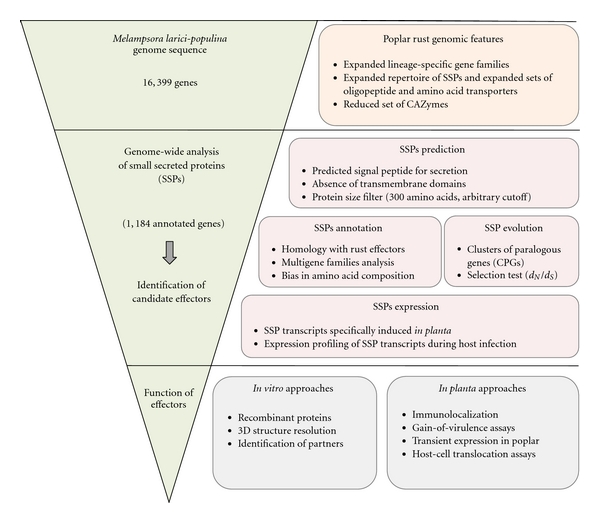
Roadmap for effectors identification in *Melampsora larici-populina*.

The genomic hallmarks reflecting the biotrophic lifestyle of the rust fungus *M. larici-populina* were recently uncovered [15]. Among the 16,399 genes reported in the poplar rust genome, a strikingly large number of expanding lineage-specific gene families were identified (909 lineage-specific gene families among 5,304 in total, corresponding to 5,798 genes). Several expanded gene families were also observed in the only other Pucciniales genome sequenced so far (the wheat stem rust* Puccinia graminis* f. sp. *tritici* [15]), including oligopeptide and amino acid transporters. Among the expanded gene families unique to the poplar rust, 54 (462 genes in total) encode small secreted proteins (SSPs) that represent putative effectors. In addition, other striking features of the poplar rust genome include a reduced set of carbohydrate active enzymes and impaired nitrogen and sulfur assimilation pathways [15]. The genomes of other plant biotrophic pathogens revealed striking similarities with rust fungi genomic hallmarks [29, 30]. A particular attention was given to the genes encoding SSPs in *M. larici-populina* (Figure 2). Indeed, many effector proteins secreted by biotrophic oomycete and fungal plant pathogens are SSPs of unknown function and their virulence or avirulence activities could determine the outcome of the interaction with the host [20, 21, 31, 32]. Consistent with the “arms race” concept between the plant immune system and pathogen effectors, SSPs could display accelerated evolution rate (i.e., positive/diversifying selection) likely to evade plant R protein-mediated recognition [33, 34]. In *M. larici-populina*, the detailed annotation of predicted SSP genes, followed by expression and adaptive evolution investigations, helped the identification of candidate rust effectors likely involved in the molecular cross-talk between the rust fungus and poplar [17, 35] (Figure 2). A total of 1,184 SSP genes have been identified and represent 7.2% of the total number of genes in the *M. larici-populina* genome [15] (Figure 2). These genes are organized in 169 gene families (the largest contains 111 gene members) dispersed in the genome, and their number supports the current view of fungal effectors as a redundant and diversified reservoir, contrasting with restricted effectors repertoire of biotrophic bacteria [36]. Interestingly, although some SSPs display similarities with effectors previously characterized in related rust species such as *Melampsora lini* (flax rust) and *Uromyces fabae* (bean rust), the majority of SSPs reported in *M. Larici-populina* (69%) are specific to this rust fungus [15, 17]. Similar observations were made after the analysis of the genome of the obligate biotrophic fungus *Blumeria graminis*, supporting the importance of clade- and lineage-specific effectors in fungi [29]. A striking feature of *M. larici-populina* SSPs is the high content of cysteine residues (63% contain more than 4 cysteines). Although the function of these residues in candidate SSP effectors is not known at the moment, it may be hypothesized that they could have a structural role through disulfide bond formation, known to stabilize proteins and enhance resistance to host proteases [37, 38]. Some of these cysteines reside in a short string of residues conserved between gene members of several SSP families ([Y/F/W]xC). Such a motif was also reported in candidate effectors of the barley powdery mildew *B. graminis* and in the wheat rust fungi *P. graminis *f. sp. *tritici* and *Puccinia triticina*, preferentially in the N-terminal region of the proteins [29, 39]. Godfrey et al. [39] proposed a possible involvement of the motif in the translocation of effectors in host plant cells, similar to the conserved N-terminal RxLR motif of oomycete effectors [40–42]. However, the [Y/F/W]xC motif is also detected in the C-terminal region of some *M. larici-populina* SSPs and is highly represented in larger nonsecreted proteins of diverse functions [15]; thus, the exact role of this motif remains to be determined. Some of the SSPs belonging to gene families were grouped in clusters of paralogous genes (CPGs) of at least three and up to 39 members with high levels of similarities [17] (Hacquard et al., unpublished data). Interestingly, the C-terminal region of SSPs of some CPGs show significant evidence of positive selection, which is strongly suggestive of a diversification of these effector-like gene families upon interaction with the host, likely to evade recognition. Such a diversification is a hallmark of biotroph effectors with avirulence functions [43–47]. Interestingly, some members of these positively selected CPGs harbour relatively well-conserved N-terminal secretion peptide as well as K/R- and D/E-rich regions (Hacquard et al., unpublished data), reminiscent of the host-cell translocation motifs reported in some fungal and oomycete effectors [40–42]. These SSPs represent potential avirulence factor for which biochemical characterisation is ongoing (Figure 2). Beyond the extensive description of the repertoire of putative effectors in the poplar rust fungus genome, such evidence of selection in paralogs argues for the use of large-scale sequencing of candidate effector genes across genus and species (i.e., isolates with distinct pathotypes) to complete the molecular landscape of effector diversity.

## 3. Poplar-Poplar Rust Transcriptomics: Insights into Plant Defense Reactions and Stage-Specific Fungal Expression Patterns

While genomic analyses reveal the genetic potential of organisms, transcriptomics allow deciphering the regulatory networks controlling the expression of such genetic programs in space and in time. In complex biotrophic systems involving two species, fine-tune genetic reprogramming occurs in both the host and the parasite to determine the outcome of the interaction [20, 48]. Indeed, plant host-specific resistance mechanisms rely on expression of inducible defense genes, whereas the biotrophic pathogen lifestyle is based on the temporal and local expression of virulence effectors in infection structures (i.e., spores, germ-tubes, invasive hyphae, haustoria). 

**Figure 3:**
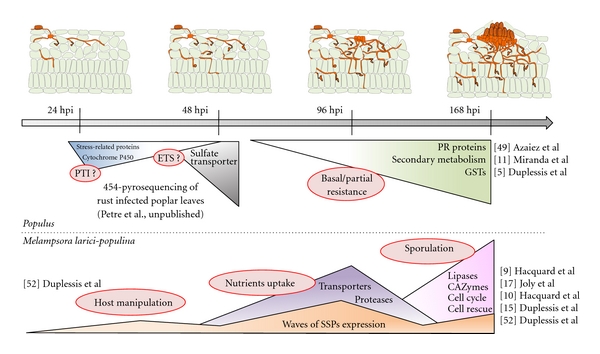
Major transcriptome regulations in a compatible *Populus/Melampsora* interaction. Triangles represent genetic programs set up by *Populus* (top) or *M. larici-populina* (bottom), and red circles indicate associated biological functions. PTI: PAMP-triggered immunity; ETS: effector-triggered susceptibility; PR: pathogenesis-related; GSTs: glutathione S-transferases; hpi: hours postinoculation, CAZymes: carbohydrate-active enzymes, SSPs: small secreted proteins.

Transcriptome studies conducted on poplar-poplar rust interactions revealed the early induction of defense responses during the incompatible interaction, referred to as complete or host-specific resistance, whereas a late induction of defense responses was observed in the case of compatible interactions and partial resistance [7, 8, 11, 49, 50] (Figure 3). These studies indicate that defense reactions are governed by common molecular bases in both perennial and annual species. Indeed, defense responses in poplar include the typical set of inducible defense genes such as PR proteins, GSTs (glutathione *S*-transferases), and redox homeostasis enzymes, as well as genes of the phenylpropanoid pathways [5]. Among the genes induced during host-specific resistance, only a few show no homology with known proteins and might represent innovations in perennial species [7, 27]. Besides, differences in poplar defense reactions set during complete and partial resistances are mainly quantitative and timely regulated [5]. Transcriptome analyses in the model plant *A. thaliana* already demonstrated the quantitative nature of differences between compatible and incompatible interactions [51]. Thus, poplar and *Arabidopsis* (and by extension perennial and annual species) seem to share a conserved set of genes to actively react upon biotroph pathogen attacks. This statement is widely applicable regarding global transcriptome analyses; however, some specificities have been described. Indeed, phylogenetically related groups of TLPs are preferentially induced in infected poplar leaves during partial or complete resistance to the rust fungus [27]. As specified above, a cluster of eleven TLP genes, so far unique to perennial species, is induced during partial resistance of poplar to rust fungi (i.e., at late infection stages of compatible interactions with *Melampsora *spp.) [27]. This regulation pattern contrasts with another clade of poplar TLPs conserved in all plants and that is mostly induced early during incompatible poplar-poplar rust interaction [27]. 

Transcriptome analyses of poplar leaves infected by either virulent or avirulent strains of *M. larici-populina* at early stages of infection (i.e., earlier than 48 hpi) are ongoing to dissect genetic reprogramming in poplar upon infection by the rust fungus (Petre et al., unpublished data). Preliminary results indicate repression of expression of genes coding for defense-related proteins and secondary metabolism enzymes at 48 compared to 18 and 24 hpi in the compatible interaction. From the “Zig-Zag” model standpoint [20], this could reflect the effector-triggered susceptibility (ETS) that promotes fungal virulence by inhibiting the plant PAMP-triggered immunity (PTI). No more than 10 genes were induced at this stage of the compatible interaction, including a dramatically induced sulphate transporter gene (Figure 3). Such an observation is puzzling since sulphate assimilation pathway seems to be impaired in rust fungi [15]. This opens interesting perspective to address the role of sulphate transport, assimilation, and metabolism in rust fungi during compatible poplar-poplar rust interactions and how it could impact host metabolism. 

*M. larici-populina* genetic programs triggered during poplar leaf infection (from urediniospore germination to uredinia formation on poplar leaves; see Figure 1(b)) have been recently investigated with whole-genome custom exon oligoarrays [9, 15, 52]. Interestingly, the set of induced fungal genes greatly differs during host colonisation with preferential transcript expression at early time points (24–48 hpi, haustoria formation), intermediate time points (48–96 hpi, biotrophic growth), and later stages (96–168 hpi, biotrophic growth, uredinia formation and sporulation) (Figure 3). Strikingly, several classes of SSP genes are successively expressed all along the infection, from germination to uredinia formation, suggesting that distinct sets of effectors are expressed to set up, promote, and maintain fungal biotrophy [52]. SSP expression during urediniospore germination or at early stages of infection (i.e., 24–48 hpi) supports an early manipulation of the plant defense system by the fungus in order to promote virulence (Figure 3). In contrast, transcripts encoding proteases and transporters were preferentially induced at 96 hpi when the fungus has already formed many haustoria in host cells, supporting the uptake of host resources and nutrients by the fungal structures as previously reported for the bean rust fungus *U. fabae* (see [53] for a complete review). At later stages of infection, the induction of transcripts encoding various lipases and carbohydrate active enzymes (CAZymes) was observed concomitantly with uredinia formation (96–168 hpi) reflecting a potential switch in lipid metabolism during the sporulation process [52] (Figure 3). Quite interestingly, four genes encoding SSPs belonging to the same gene family (*M. lini *HESP-417 homologs) were expressed at different infection stages, and their expression profiles were confirmed by RT-qPCR [52]. This result suggests that waves of expression of SSP genes could likely contribute to the turnover/renewal of SSPs at the interface with the host or inside host cells during the biotrophic interaction. 

In transcriptomic studies of biotrophic interactions, isolation of transcripts from infected tissues does not give access to specific patterns of gene expression in distinct pathogenic structures. The purification of haustoria from rust-infected bean leaves has paved the way for the molecular analysis of these fungal cell types that play a key role in the establishment of obligate biotrophy. Such an approach has allowed the sequencing of haustoria-specific cDNA and the identification of genes expressed in these infection structures [53–56]. This approach was successfully used to identify several candidate effectors in haustorially expressed secreted proteins (HESPs) of the model rust fungus *M. lini* [57], including several avirulence genes. However, specific *in planta *gene expression in other cell types (i.e., infection hyphae, sporogenous hyphae) was not assessed so far in rust fungi. Isolation of biotrophic infection hyphae from *Colletotrichum higginsianum *was successfully applied to *Arabidopsis* infected leaves for transcriptome analysis [58], and rust infection hyphae and derived infection structures were obtained artificially for the bean rust fungus *U. fabae* (see [53] for details). The use of laser capture microdissection (LCM) was a major breakthrough to dissect the genetic programs related to biotrophy and sporulation at a late stage of rust infection (168 hpi), whereas various fungal structures are formed *in planta* [9]. LCM allowed the successful isolation of infected mesophyll tissues containing infection hyphae and haustoria, as well as uredinia containing sporogenous hyphae and newly formed urediniospores for downstream expression analysis using *M. larici-populina* whole-genome custom exon oligoarrays [9]. Strikingly, among the most highly induced transcripts detected in fungal biotrophic structures in the LCM-isolated palisade mesophyll (>100-fold induction compared to LCM-isolated uredinia), almost all encode SSPs. This unexpected high number of candidate effectors expressed at this late stage of rust infection might be essential for the maintenance of biotrophy during uredinia formation and sporulation. Preventing structures like haustoria from host detection in photosynthetically active mesophyll might be crucial to preserve the capability of the rust fungus to extract nutrients from the plant toward sporulating structures. Indeed, in nature, sporulation structures are maintained in infected leaves and can produce thousands of urediniospores over several weeks. Transcripts induced in LCM-isolated uredinia are more diverse and include several cell-cycle- and cell-rescue-related transcripts [9]. The induction of cell cycle transcripts is supportive of the intense cell division activity observed in the microdissected sporulation area, while cell-rescue- and defense-related transcripts might reflect an active defense from the rust fungus in response to the late activation of poplar reactions such as oxidative burst or expression of defense genes reported in compatible poplar-poplar rust interactions [5, 8] (Figure 3). Interestingly, transcripts accumulated in the sporulation area were predominantly detected in resting urediniospores and at 168 hpi in the time-course expression study [52]. In contrast, genes induced in the microdissected-infected mesophyll and encoding SSPs were predominantly expressed at 96 hpi during the time-course infection, at a stage that only consists in haustoria and infection hyphae [52]. Such observations support a transcriptional switch between different fungal cell types in infected plant tissues at the stage of uredinia formation and sporulation.

The combination of tools like transcriptomics and LCM clearly demonstrates that a deeper and comprehensive view of rust fungi genetics can be gained from the study of *in planta* infection structures. Future directions will concern the identification of the fungal determinants that control the switch leading to uredinia formation in the host and the molecular bases controlling biotrophy.

## 4. Toward Candidate Effector Characterisation: The Need for Functional Approaches

Combination of genome and transcriptome analyses led to the definition of a very large repertoire of candidate* M. larici-populina *effectors [15, 17]. Ongoing studies based on high-throughput genome and transcriptome sequencing of additional poplar rust isolates with defined pathotypes will complete and precise the list of candidate effectors. Thus, the step defined as “effector discovery” by Alfano [59] is rather advanced and almost complete, with an exhaustive inventory of putative effectors for this rust fungal pathogen. The next step will consist in the characterisation of effector virulence/avirulence functions (Figure 2). These functional investigations will be based on the heterologous expression of candidate effector coding sequences in *Escherichia coli* or other production systems for subsequent purification of recombinant proteins. Protein expression patterns, subcellular localisation, biochemical features analyses, and resolution of tridimensional structures as well as protein-protein interaction assays will help to address major questions concerning the role(s) of the highly diverse and redundant set of SSP genes in virulence and biotrophy. Are some effectors translocated into host cells? How do cytoplasmic effectors enter host cells? Where do they localize and is this localisation dynamic upon infection? What are the targets or interactors of fungal effectors in host cells and how do they interact? In order to have an accurate vision of effector functions, putative effector targets should be analyzed in a similar way [59]. Beyond *in vitro* analyses, *in vivo* experiments are also required to validate the effector actions on the host. Many efforts are currently underway to establish assays for genetic transformation in poplar and/or in *Melampsora* to study *in vivo* the function of genes involved in the poplar-poplar rust interaction. Characterisation of virulence function of *M. larici-populina *effectors using a bacterial-based protein injection system in *Arabidopsis* is currently in development (H. Germain and A. Séguin, personal communication). In this system, bacterial growth inside plant leaves is used as a reporter of effector-triggered plant defense inhibition [60]. In addition, transient expression assays of effector proteins in poplar leaves could lead to the characterisation of avirulence functions reported by localized hypersensitive response. Molecular analyses of the flax-flax rust pathosystem represent the most advanced rust pathosystem in the field [57, 61–63] and is inspiring to set strategies toward functional characterisation of poplar rust effectors and avirulence genes. The large range of hybrid poplars harbouring up to eight complete resistances constitutes a robust basis for such future screening experiments.

## Figures and Tables

**Table 1:** Summary of genome-wide studies conducted on disease-related gene families in poplar.

Gene family analyzed	References	Implication in plant defense reactions	References
*NB-LRR *(nucleotide-binding leucin-rich repeat)	Kohler et al. [19]	Gene-for-gene resistance mechanisms, host-specific resistance (R proteins)	Jones and Dangl [20]
*BED-NB-LRR * (BED family of poplar NB-LRR)	Kohler et al. [19] Germain and Séguin [24]	BED domain is a zinc-finger DNA-binding domain	Markljung et al. [64]
*TLPs* or PR5 (thaumatin-like proteins)	Petre et al. [27]	Antimicrobial and glycan-degrading activities	Liu et al. [65]
*Phenylpropanoid* metabolism (secondary metabolism)	Tsai et al. [66] Hamberger et al. [67]	Phytoalexin synthesis and cell wall reinforcement upon pathogen attack	Bednarek and Osbourn [68]
*CAD * (cinnamyl alcohol dehydrogenase)	Barakat et al. [69]	Lignin biosynthesis, cell wall reinforcement upon pathogen attack	Tronchet et al. [70]
*YUCCA *(auxin biosynthesis)	Ye et al. [71]	Auxin can promote virulence during biotrophic infection	Grant and Jones [72]
*ARF and Aux/IAA * (auxin-response transcription factor)	Kalluri et al. [73]	Auxin can promote virulence during biotrophic infection	Grant and Jones [72]
*AP2/ERF *(Ethylene-response transcription factors)	Zhuang et al. [74]	Regulation of disease resistance pathways	Gutterson and Reuber [75]
*R2R3MYB * (transcription factors)	Wilkins et al. [76]	Regulation of secondary metabolism (in Response to pathogen attack)	Mellway et al. [77]
*LysM kinase * (signal transduction)	Zhang et al. [78]	Chitin signaling and fungal resistance	Wan et al. [79]
*PLD * (phospholipase D)	Liu et al. [80]	Secondary messenger release upon pathogen attack	Wang [81]
*Protease * (protein degradation)	Garcia-Lorenzo et al. [82]	(Pathogen) protein degradation, regulation of plant cell death	Solomon et al. [83]
*F-box * (selective degradation of proteins)	Yang et al. [84]	Pathogen effector targets for host manipulation	Block et al. [85]
*Kunitz-trypsin inhibitors * (protease inhibition)	Major and Constabel [86]	Inhibition of pathogen protease and pathogen-triggered cell death	Li et al. [87]
*RBP * (RNA binding proteins)	Peal et al. [88]	Pathogen effector targets for host manipulation	Fu et al. [89]
*GST * (glutathione transferase)	Lan et al. [90]	Xenobiotic detoxification and redox homeostasis	Dixon et al. [91]
*Grx * (glutaredoxin)	Couturier et al. [92]	Redox metabolism and homeostasis controlling oxidative burst	Rouhier et al. [93]
